# A Misleading Neck Mass: Branchial Cleft Cyst Mimicking Anaplastic Thyroid Carcinoma

**DOI:** 10.1210/jcemcr/luaf201

**Published:** 2025-09-19

**Authors:** Taylor L Jamil, Ryan Thomas, Christie G Turin, Brian P Cervenka, Nikita Pozdeyev

**Affiliations:** Department of Otolaryngology, University of Colorado School of Medicine, Aurora, CO 80045, USA; Department of Pathology, University of Colorado School of Medicine, Aurora, CO 80045, USA; Division of Endocrinology, Diabetes, and Metabolism, University of Colorado School of Medicine, Aurora, CO 80045, USA; Department of Otolaryngology, University of Colorado School of Medicine, Aurora, CO 80045, USA; Division of Endocrinology, Diabetes, and Metabolism, University of Colorado School of Medicine, Aurora, CO 80045, USA; Department of Biomedical Informatics, University of Colorado School of Medicine, Aurora, CO 80045, USA

**Keywords:** anaplastic thyroid cancer, branchial cleft cyst, neck mass, lymph node dissection

## Abstract

A 33-year-old female individual had a history of a lymph node excision positive for metastatic papillary thyroid carcinoma (PTC) in 2016. She was treated with a total thyroidectomy and central neck dissection showing 0.2-cm micro-PTC without paratracheal lymph node involvement, followed by adjuvant radioactive iodine treatment with 104 mCi of iodine-131. After 9 years without evidence of disease, she presented with a mildly tender, firm, left neck level II mass rapidly enlarging over 3 weeks. Neck ultrasound revealed a 3.5 cm irregular hypoechoic mass with internal vascularity and punctate echogenic foci. A fine needle biopsy showed inflammatory and mature squamous cells with elevated thyroglobulin but no definitive evidence of malignancy. The patient was scheduled for urgent neck dissection; however, just a left lymph node excision was performed as intraoperative frozen pathology showed no cancer. Surgical histopathology showed branchial cleft cyst tissue. The most concerning diagnosis for a rapidly enlarging neck mass in an adult with a history of metastatic PTC is anaplastic thyroid carcinoma. However, other diagnoses such as branchial cleft anomalies, lymphoma, or non-thyroid metastatic malignancies should be considered. Intraoperative frozen section pathology is crucial to direct surgical management of an unknown neck mass concerning for anaplastic thyroid cancer.

## Introduction

Rapidly enlarging cervical masses, in the absence of trauma or recent surgery, are often due to infection or malignancy. Specifically, in a patient with a history of metastatic papillary thyroid carcinoma (PTC), lymphoproliferative disorders, aggressive differentiated thyroid cancer, and anaplastic thyroid cancer (ATC) are important to rule out [1].

ATC is a rare, highly aggressive, undifferentiated form of thyroid cancer that arises from follicular thyroidal cells [2]. ATC encompasses 1% to 3% of all patients with thyroid cancers [3, 4]. About 60% of cases are female, with most patients being diagnosed in the sixth and seventh decades of life [3]. Patients with ATC have a poor prognosis, with a median overall survival of 3 months [4].

Branchial cleft anomalies are common pediatric neck masses; however, the exact population prevalence is unknown (Table 1) [5]. They are caused by incomplete obliteration of embryological remnants of the branchial apparatus. Most cases are detected in the first decade of life [6]. Second branchial cleft cysts are the most common [7]; however, branchial cleft anomalies are far less studied in adults due to their rarity.

**Table 1.** Branchial cleft anomaly subclassification and anatomic location

Branchial cleft anomaly	Location
Type I Work Type I and II	Periauricular in location in which the cyst tract opens in the external auditory canal. Work type I cysts end periauricularly. Work type II anomalies are often associated with the parotid gland a facial nerve.
Type II	Connected to the pharynx, often around the tonsillar fossa. Found along the anterior border of the sternocleidomastoid muscle, lateral to the carotid sheath and posterior to the submandibular gland.
Type III	Connected to the pharynx, often around the tonsillar fossa. Often extend medially between the bifurcation of the external and internal carotid arteries. Anterior to the sternocleidomastoid muscle.
Type IV	Often forms a fistula/sinus tract around the larynx and is medial to the carotid sheath.

We present a case of an adult female patient with a history of metastatic PTC who developed a rapidly enlarging left level II neck mass suspicious for ATC, which was found to be a branchial cleft cyst.

## Case Presentation

A 33-year-old female individual had a history of metastatic PTC diagnosed after excision of a single, palpable, cervical lymph node in 2016. She underwent a total thyroidectomy and central neck dissection, which revealed a unifocal 0.2-cm classic micro-PTC and 2 perithyroidal lymph nodes negative for cancer (pT1aN1bM0) [8]. She received 104 mCi of radioactive iodine (^131^I) as an adjuvant therapy. This treatment was performed at an outside hospital, and the detailed pathologic features or the rationale for ^131^I therapy are unknown. Due to the presence of palpable nodal disease (clinical N1 thyroid cancer), her risk of structural disease recurrence (RoR) is intermediate (∼20%), which may justify radioactive iodine treatment [8].

She had no evidence of thyroid cancer recurrence until 9 years later, when, over a period of 3 weeks, she developed a rapidly enlarging left level II neck mass mildly tender to palpation. She noted increasing fatigue and mild upper respiratory symptoms but had no fever, voice changes, or dyspnea.

## Diagnostic Assessment

**Figure 1.**
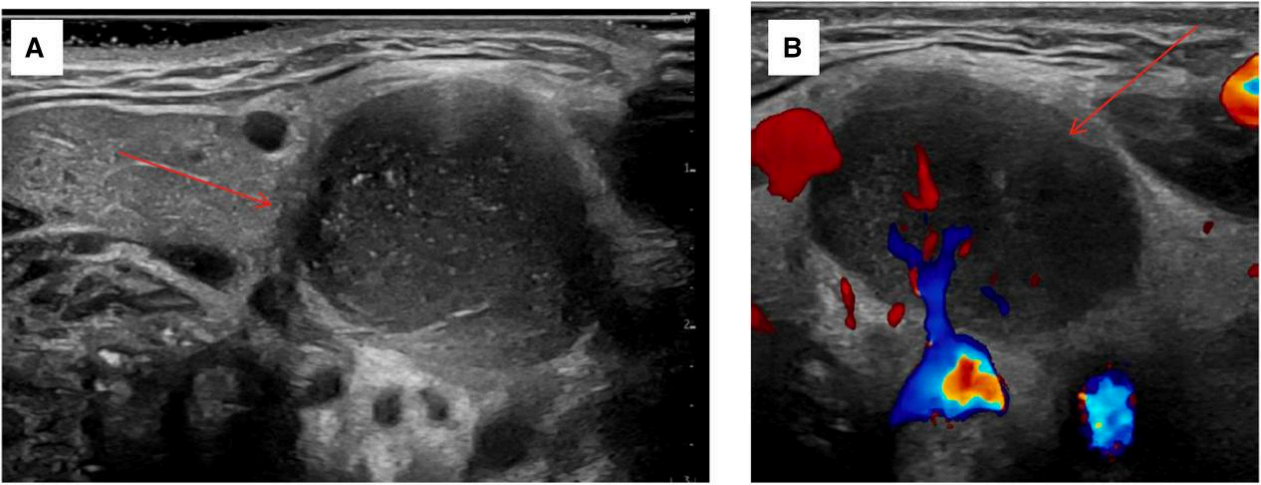
Ultrasound transverse (A) and color Doppler (B) view of the left level II nodule in a young female patient with a history of papillary thyroid cancer without evidence of disease for about 9 years who presented with a rapidly enlarging left neck mass over a 3-week timespan.

**Figure 2.**
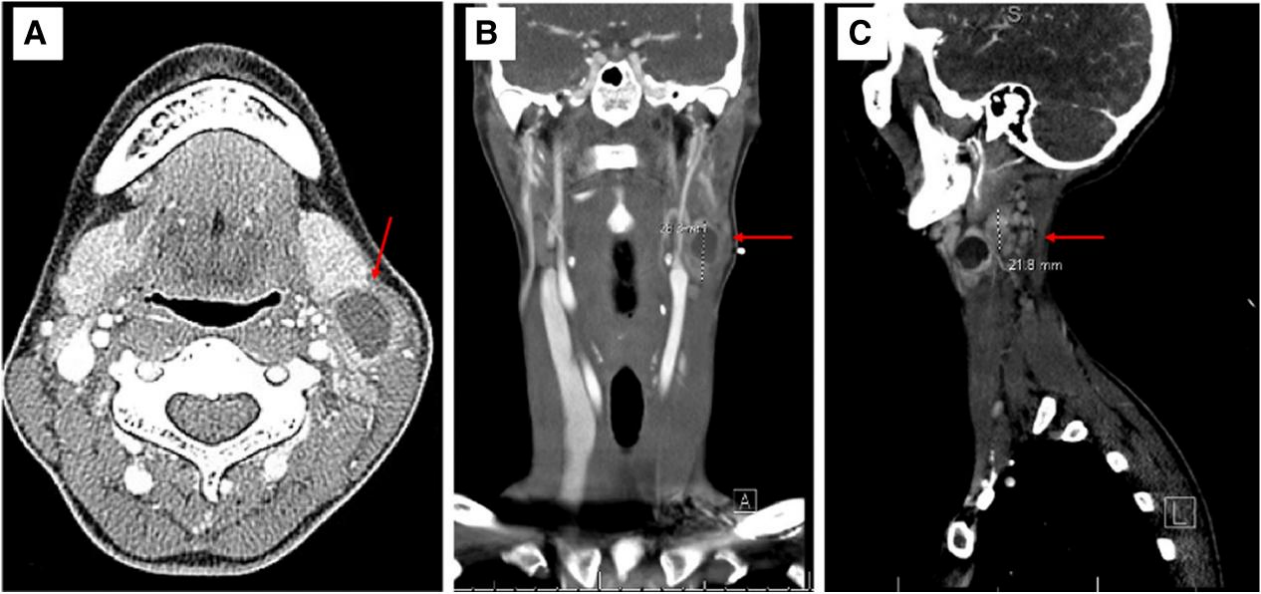
Computed tomography (CT) of the neck with contrast in axial (Photo A), coronal (Photo B), and sagittal (Photo C) plane images of the left level II neck mass in a young female with a history of papillary thyroid cancer who presented with a rapidly enlarging left neck mass. The radiology report showed “left cervical adenopathy with dominant 18 × 18 × 28 mm necrotic zone 2 node with irregular borders. Multiple adjacent smaller nodes. Differential is metastatic adenopathy. Infected reactive adenopathy considered less likely given morphology.”

The patient presented to her primary care physician, who noted a firm 3-cm mass, fixed to the surrounding tissues. Blood tests showed a mildly increased C-reactive protein, but otherwise normal inflammatory serum markers (Table 2). Neck ultrasound showed a new left zone II hypoechoic mass with internal vascularity and punctate echogenic foci measuring 3.5 × 2.2 × 1.8 cm (Fig. 1) and a new left zone VI hypoechoic nodule with internal vascularity, questionable punctate echogenic foci but preserved vascular hilum measuring 0.6 × 0.4 × 0.4 cm (not shown). Computed tomography (CT) of the neck found left cervical adenopathy with a dominant 1.8 × 1.8 × 2.8 cm mass described in the radiology report as a necrotic zone 2 mass with irregular borders and multiple adjacent smaller nodes (Fig. 2). The radiologist interpreted these findings as likely metastatic adenopathy due to her medical history, and reactive adenopathy of an infectious cause was considered less likely.

**Table 2.** Laboratory test results for a patient with a history of T1N1M0 micro-PTC with a rapidly enlarging 3-cm left level II neck mass

Parameter	Test result, conventional and (SI) units	Reference range, conventional and (SI) units
C-reactive protein	1.05 mg/dL (SI: 10.5 mg/L)	0-0.5 mg/dL (SI: 0-5 mg/L)
White blood cells	SI: 4600 cells/mm^3^ (SI: 4.6 × 10^9^/L)	3800-9800 cells/mm^3^ (SI: 4.0-11.1 × 10^9^/L)
Erythrocyte sedimentation rate	2 mm/h (SI: 0-20 mm/h)	0-20 mm/h for females (SI: 0-20 mm/h)
Lactate dehydrogenase	123 U/L (SI: 2.05 μkat/L)	100-190 U/L (SI: 1.67-3.17 μkat/L)
Thyroid-stimulating hormone	1.99 mIU/L (SI: 1.99 mIU/L)	0.45-5.33 mIU/L (SI: 0.45-5.33 IU/L)
Blood serum thyroglobulin	2.2 ng/mL (SI: 2.2 µg/L)	Female 12 years and older: 0.5-43.0 ng/mL (SI: 0.5-43.0 μg/L)
Thyroglobulin antibody	< 1 IU/mL (SI: < 1 µg/L)	<4 IU/mL (SI: < 4 µg/L)
Thyroglobulin washout from FNA biopsy	2.2 IU/mL (SI: 2.2 IU/mL)	≤ 1 IU/mL (SI: ≤ 1 IU/mL)

Abbreviations: FNA, fine needle aspiration; PTC, papillary thyroid carcinoma.

The following day, she underwent ultrasound-guided fine needle aspiration (FNA) of the left level II mass. Thyroglobulin washout was mildly elevated at 2.2 ng/mL (2.2 ng/mL) (normal reference range: ≤1 ng/mL; SI: ≤1 ng/mL). The FNA showed abundant inflammatory and squamous cells with rare cells with nuclear atypia. The patient had normal serum thyroid-stimulating hormone, low serum thyroglobulin, and undetectable serum thyroglobulin antibody levels (Table 2). FNA material was sent for bacterial and fungal cultures, which were negative. Due to the cystic nature of the lesion and the low cellularity of the specimen, molecular diagnostic testing was not performed. With the patient's history of metastatic PTC, ATC was suspected, although other etiologies such as non-thyroidal metastatic disease, lymphoma, infection, or a benign mass were considered. Due to her history of thyroid cancer with intermediate RoR, irregular borders of the neck mass on CT scan, the radiologists’ concern for malignancy, and inconclusive results from the FNA, a surgical excision was pursued.

## Treatment

The patient was empirically treated with amoxicillin/clavulanate 875/125 mg twice daily for 7 days for an infected cyst without clinical improvement. Core biopsy was considered but not performed because, she was scheduled for urgent left level 2 mass excision. Intraoperatively, extensive scarring, likely due to chronic cyst infection and prior surgery in the neck, was found. However, the mass was successfully dissected away from the internal jugular vein and accessory nerve. Intraoperative frozen section pathology was consistent with a benign epithelial-lined cyst; therefore, the procedure was terminated, and complete neck dissection was not performed.

She recovered well postoperatively, with minimal accessory nerve weakness and mild difficulty raising her arm above her shoulder, which resolved over a month. No further treatment was needed.

## Outcome and Follow-Up

**Figure 3.**
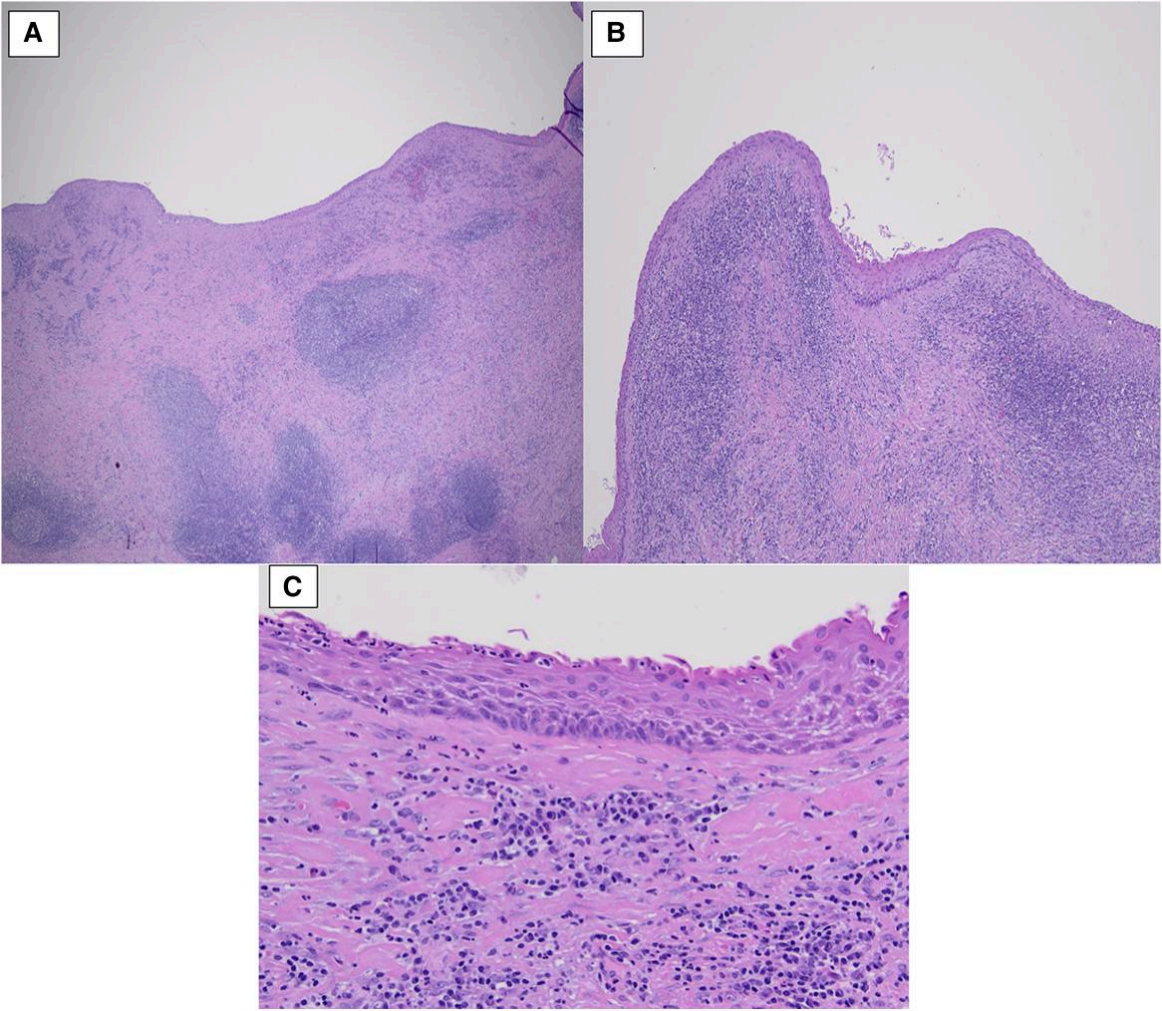
Photos A–C: Photo A and B, Low-power views of the excision specimen show a unilocular cyst lined by stratified squamous epithelium with prominent lymphoid tissue in the cyst wall after surgical resection of the left neck mass from a young female patient with a rapidly enlarging left neck mass with a history of papillary thyroid cancer (Photo A: hematoxylin and eosin, 40× magnification. Photo B: hematoxylin and eosin stain, 20× magnification.). Photo C, Histopathological section in a high-power view of the excised specimen shows intraepithelial and intramural acute inflammation after surgical resection of the left neck mass. No evidence of malignancy was seen. The histomorphologic findings are consistent with an inflamed/ruptured branchial cleft cyst. (hematoxylin and eosin stain, 200× magnification).

Histopathologic preparations of the left neck mass excision specimen demonstrated abundant mature squamous cells in a background of mixed inflammatory cells and amorphous keratinous debris (Fig. 3A). Rare cells with mild cytologic atypia were identified and were favored to represent reactive branchial cleft cyst contents vs possible neoplastic cells. No overt evidence of malignancy was seen and immunohistochemical stain for p16 performed on the cell block preparation was negative. Frozen section and histologic evaluation of the excised specimen demonstrated a unilocular cystic lesion lined by stratified squamous epithelium with scattered intraepithelial acute inflammation and no features concerning for malignancy (Fig. 3B). The cyst wall contained prominent lymphoid tissue with admixed acute inflammatory cells. No evidence of thyroid carcinoma was seen. The findings were consistent with an inflamed/ruptured branchial cleft cyst (Fig. 3C).

## Discussion

We present a case of an adult female patient with an enlarging neck mass. Surgical pathology found a congenital branchial cleft cyst anomaly. Because of her prior history of metastatic PTC, her treatment team was concerned with anaplastic transformation of cancer cells. FNA cytology was not consistent with hematologic malignancy. Congenital abnormalities, such as an infected branchial cleft cyst, were considered, and she was treated empirically with amoxicillin/clavulanate. However, people older than their first to second decade of life rarely present with congenital abnormalities. As there was detectable thyroglobulin, albeit minimally elevated, in the FNA needle wash and negative bacterial culture in FNA contents, the diagnosis of ATC was entertained [9]. Also, she had no history of a fluctuating, painless mass, as is usual for branchial cleft cyst anomalies [10].

ATC is a rare but aggressive form of dedifferentiated thyroid cancer with a median overall survival of about 3 months [11, 12]. No significant risk factors were identified for ATC. It is proposed that ATC originates from differentiated thyroid cancers in a process called anaplastic transformation [13, 14]. FNA is the standard initial procedure when diagnosing ATC [12]. As ATC cells may look vastly dissimilar to non-cancerous thyroid tissue, diagnosing ATC using FNA is sometimes difficult. It was previously shown that ATC can be diagnosed with FNA about 60% of the time; however, there is high inter-observer bias, and the diagnostic yield is lower in patients with less cellular, more cystic nodules [1]. Including FNA samples from different regions within the mass can also be helpful. If differentiated thyroid cancer is found next to dedifferentiated cells, the diagnosis is established.

It is unclear why the thyroglobulin wash performed from the FNA was elevated in our patient. Possible explanations are a laboratory error, a test-related false positive result, or that the patient has occult recurrent thyroid cancer that was not visible on imaging. Ectopic thyroid tissue in branchial cleft cysts, including isolated PTC, has been described [15]. However, histopathological review did not reveal any thyroid tissue in our patient. A study by Kargun et al (2023) evaluated thyroglobulin washes and found a sensitivity of 91.8% and a specificity of 96.6% in detecting malignant lymph nodes [16].

ATC is heterogeneous, with spindle cell, giant cell, squamous, and epithelial subtypes described [17]. Squamous cells on FNA were concerning for the squamous ATC subtype. Additionally, elevated inflammatory markers can be seen in both ATC and infectious processes. When the diagnosis of ATC via FNA is more difficult, surgical biopsy and/or resection can provide a definitive diagnosis [1, 8]. The current ATC clinical practice guidelines recommend frozen section analysis intraoperatively if open surgical resection is performed [8]. Lymphoma workup can also be added to intraoperative analysis [12].

Branchial cleft anomalies result from a persistent branchial apparatus that fails to obliterate during embryological development. Anomalies present as either cysts, sinuses, or fistulas. All branchial cleft anomalies are lined with stratified squamous epithelium and may contain keratinous debris [6]. There are 4 subtypes of branchial cleft cysts; the most common is a second branchial cleft anomaly [5]. Type II-IV branchial cleft cysts often present in the lateral neck anterior to the sternocleidomastoid muscle and their relationship to other arteries and musculature depends on the subtype. Second branchial cleft cysts usually drain into the tonsillar fossa and course between the internal and external carotid, ending in a cyst wall in the middle of or anterior to the sternocleidomastoid muscle (Table 1). A case series by Ahuja et al (2000) found that in adults, branchial cleft cysts can look more heterogenous than classically described in the pediatric population [16]. Branchial cleft cysts can change in size due to upper respiratory infections and other pro-inflammatory conditions and are usually diagnosed during childhood [16]. The true incidence of branchial cleft cysts identified after the age of 18 is unknown due to their rarity. Kajosaari et al (2014) found that 24% of patients had an infected branchial cleft cyst on initial diagnosis, but the majority presented with a painless fluctuating mass [18]. Once excised, recurrence is rare, at about 3% [6].

In a young adult, type II branchial cleft cysts are rare malformations. In our patient with a history of PTC, benign diagnoses were considered, but the concern for ATC remained high. Previous operations in the lateral neck space, suspicious imaging findings, and ambiguous test results made her diagnosis unclear until excisional biopsy established a definitive diagnosis of a branchial cleft cyst. Multidisciplinary care and real-time decision-making in the operating room facilitated by intraoperative frozen section analysis ensured optimal management.

## Learning Points

- The benign etiology of a rapidly growing neck mass should be considered, even in patients with a prior history of metastatic thyroid cancer.
- Differentiating ATC from other neck masses is challenging, and urgent excisional biopsy may be considered.
- Intraoperative frozen section pathology evaluation of the excised neck mass is helpful to guide the extent of the surgery.

## Contributors

All authors made individual contributions to the manuscript. N.P., C.G.T., and B.P.C. were involved with the diagnosis and management of the patient. T.L.J. was involved with obtaining patient consent and writing the manuscript. R.T. was involved with reviewing, interpreting, and formatting histopathology information in the manuscript and figures. All authors reviewed and approved the final draft.

## Data Availability

Original data generated and analyzed during this study are included in this published article.

## Abbreviations

ATC: anaplastic thyroid cancer
CT: computed tomography
FNA: fine needle aspiration
PTC: papillary thyroid carcinoma
RoR: risk of recurrence
